# Superresolution and Fluorescence Dynamics Evidence Reveal That Intact Liposomes Do Not Cross the Human Skin Barrier

**DOI:** 10.1371/journal.pone.0146514

**Published:** 2016-01-11

**Authors:** Jes Dreier, Jens A. Sørensen, Jonathan R. Brewer

**Affiliations:** 1 Advanced bioimaging group/MEMPHYS Center for membrane biophysics, Department of Biochemistry and Molecular Biology, University of Southern Denmark, Odense, Denmark; 2 Department of reconstructive surgery, Odense University Hospital, Odense, Denmark; University of Helsinki, FINLAND

## Abstract

In this study we use the combination of super resolution optical microscopy and raster image correlation spectroscopy (RICS) to study the mechanism of action of liposomes as transdermal drug delivery systems in human skin. Two different compositions of liposomes were applied to newly excised human skin, a POPC liposome and a more flexible liposome containing the surfactant sodium cholate. Stimulated emission depletion microscopy (STED) images of intact skin and cryo-sections of skin treated with labeled liposomes were recorded displaying an optical resolution low enough to resolve the 100 nm liposomes in the skin. The images revealed that virtually none of the liposomes remained intact beneath the skin surface. RICS two color cross correlation diffusion measurements of double labeled liposomes confirmed these observations. Our results suggest that the liposomes do not act as carriers that transport their cargo directly through the skin barrier, but mainly burst and fuse with the outer lipid layers of the stratum corneum. It was also found that the flexible liposomes showed a greater delivery of the fluorophore into the stratum corneum, indicating that they functioned as chemical permeability enhancers.

## Introduction

Successful *transdermal drug delivery* is hindered by the inability of most drugs to penetrate the skin barrier at therapeutically beneficial rates [1, 2]. Unaided, very few drugs can cross the skin barrier, and therefore permeability enhancers are needed in order to facilitate transdermal delivery. Consequently, to advance the development of efficient permeability enhancers, a thorough knowledge of each enhancer’s efficiency and mechanism of action is needed. Several techniques have been applied including microneedles [3], iontophoresis [4], sonophoresis [5], and lipid vesicle carriers.[6, 7]

One of the lipid vesicle candidates for transdermal drug delivery is liposomes or large unilamellar vesicles (LUVs). They are widely used in the cosmetic industry and have been proposed as vehicles to transport and deliver drugs through the skin barrier [7–9]. Many studies have shown that so-called flexible LUVs function as superior transdermal penetration enhancers [10]. However, despite intensive research, the mechanisms by which liposome systems deliver drugs into intact skin are still not fully understood [6, 11]. Several different techniques have been used to document the penetration of liposomes into the skin, including scanning electron microscopy (SEM) and transmission electron microscopy (TEM) [12–15], tape stripping [14], laser scanning confocal (LSM) [9, 16, 17]and multi photon excitation fluorescence microscopy (MPEM) [18, 19], all without conclusively demonstrating how the liposomes facilitate transdermal drug delivery [9, 17, 20–22]. Recently we showed, using a combination of cross correlation raster image correlations spectroscopy (CC-RICS) [23] and MPEM imaging, that the LUVs are destabilized and lose their content upon contact with the skin [24]. However, the question remains whether entire vesicles can pass into the skin or if they are broken down and merge with the SC lipids.

Confocal fluorescence microscopy studies have exploited co-localization analysis of two-color fluorescently labeled liposomes [17] to detect liposome penetration into the skin. However, considering that the average size of the liposomes are in the order of 100 nm in diameter, which is well below the resolution of the optical microscope, co-localization studies of two-color fluorescently labeled liposomes in optical microscopy are insufficient to prove the presence of intact liposomes penetrating into the skin. CC-RICS can be used to detect if two different fluorescent species of molecules diffuse together [23, 24]. Thus, this dynamic information can circumvent the diffraction barrier and provide conclusive information to predict whether intact liposomes can penetrate the epidermis or if they are destabilized and burst during transdermal penetration.

Since the early 1990s work has been done on different methods to overcome the diffraction limit of 200–300 nm in optical microscopy. Stimulated emission depletion (STED) microscopy is a promising super resolution technique, developed by the group of Stefan Hell [25, 26]. It has been demonstrated to have an optical resolution down to 20 nm in cells and down to 40 nm in tissue [27] and can be used in live samples and intact tissue specimens. This is in contrast to other higher resolution imaging techniques such as electron microcopy, which require extensive sample preparation. Thus STED can be used to bridge the gap between normal optical microscopy and electron microscopy. Seeing as the size of the LUVs and the lipid layers in extracellular space of the stratum corneum (SC) are in the order of 100nm, STED microscopy can be used to resolve these structures and the individual vesicles.

In this paper we employ STED and CC-RICS to further investigate the mechanism of action of vesicles as permeability enhancers. Specifically, we have investigated the penetration of POPC (1-palmitoyl-2-oleoyl-sn-glycero-3-phosphocoline) LUVs and a flexible vesicle (FLUVs) in human skin using CC-RICS of two color fluorescently labeled liposomes and STED microscopy to investigate whether the intact vesicles penetrate into the skin or if they break upon contact with the skin.

## Materials and Methods

### Materials

Lissamine-rhodamin B 1,2-dihexadecanoyl-*sn*-glycero-3-phosphoethanolamine (RhB-PE) was purchased from Invitrogen (Copenhagen, Denmark). ATTO-647N-PE and ATTO-488-DPPE (ATTO-488 1,2-dipalmitoyl-sn-glycero-3-phosphoethanolamine) was purchased from ATTO-TEC GmbH (Siegen, Germany). 1-palmitoyl-2-oleoyl-sn-glycero-3-phosphocoline (POPC) and Soy PC (catalog #840054) were from Avanti Polar Lipids (Alabaster, AL). Sodium cholate and all other chemicals used were obtained from Sigma-Aldrich (Copenhagen, Denmark). The buffer used was 10 mM phosphate buffered saline (PBS), 137 mM NaCl, and 2.7 mM KCl, pH 7.4.

### Skin

Human skin samples were obtained from operations on breast reduction and abdominoplasty. Non-colored ethanol iodide was used for disinfection. The samples were processed 2–24 hours after surgery, and were stored at 4°C until processing. The experiments performed in this work, involving the use of human samples, were approved by the Regional Research Ethics Committee of Southern Denmark, and were adherent to the Declaration of Helsinki Principles (2008). The requirement for written informed consent from the participants was waived by the Regional Research Ethics Committee of Southern Denmark, because Danish regulations consider human tissue left over from surgery as discarded material.

The skin was trimmed into 0.5–1 mm-thick 1 cm^2^ sections, washed in tap water, patted dry on the surface, and placed with the SC side up on a PBS-wetted filter paper.

### Liposome (LUV) and flexible liposome (FLUV) preparation

LUV: POPC stock (10mM) dissolved in 2:1 (vol/vol) chloroform/methanol was transferred with or without dyes to a glass tube and dried under nitrogen followed by hydration in PBS buffer by vortex mixing. FLUVs were prepared as described earlier [17, 20, 24]. Briefly, Soy-PC was dissolved in 2:1 (vol/vol) chloroform/methanol mixture and an appropriate amount was transferred to a glass tube with or without dyes and dried under nitrogen. The mixture was dissolved in PBS at pH 7 with an appropriated amount of sodium cholate. The soy PC and sodium cholate was mixed 87:13 w/w.

The dyes used were 0.5 mol% Atto-488-DPPE for STED imaging or a combination of 0.2 mol% RhB-PE and 0.02 mol% ATTO-647N-PE for the RICS measurements. See Table 1. The final lipid concentration was 2 mM for the labeled (F)LUV and 10mM/20mM for the unlabeled (F)LUVs. The resulting mixture of large multilamelar vesicles were extruded 20 times through a stack of polycarbonate filters of 0.1-mm pore size to produce liposomes at about 100±10 nm diameter.

**Table 1:** The dyes used for labeling the liposomes in the STED and RICS experiments.

	Dyes used
RICS	0.2 mol% RhB-PE and 0.02 mol% ATTO-647N-PE
STED	0.5 mol% Atto-488-DPPE for STED

### Labeling of skin samples with LUVs or FLUVs

Each sample was labeled with 40 μL LUVs or FLUVs solution per cm^2^ skin surface and incubated non-occlusively (i.e. the buffer containing the liposomes on the SC surface was allowed to evaporate) for 4–8 hours, at least until the surface was visually dry. The samples for cryo embedding were left overnight (12 hours). Samples were labeled with LUVs and FLUVs for three different experiments: RICS measurements, STED imaging of intact skin and STED imaging on frozen skin sections. For the RICS measurements, the combination of RhB-PE and Atto-647N-PE was used and the labeled LUVs and FLUVs were diluted 1:1000 (vol:vol) with unlabeled LUVs and FLUVs respectively. After labeling the samples for RICS were mounted on top (dermis side down) of a piece of PBS-wetted filter paper on a microscope slide and a vapor-wetted coverslip was mounted on top of the SC. The two STED experiments were done with LUVs and FLUVs labeled with ATTO-488-DPPE. Those samples were not diluted with unlabeled LUVs and FLUVs. The samples designated for cryosectioning were embedded in Tissue-Tek^™^ immediately after the incubation.

### Cryo embedding and sectioning of samples

**Fig 1:**
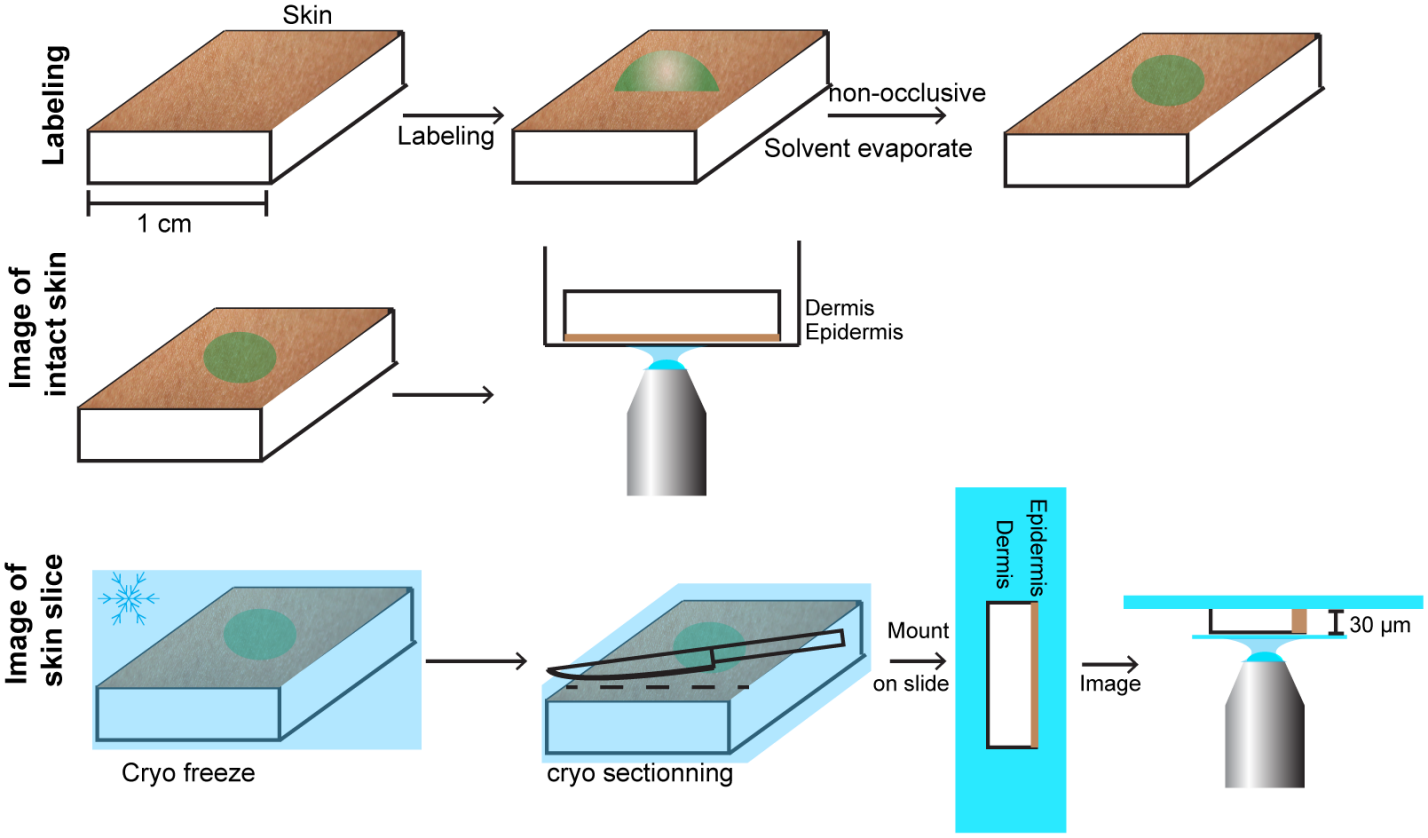
Cartoon depicting the sample preparation. Samples were labeled non-occlusively. Intact skin samples for imaging were mounted SC side down on a microscope cover glass for imaging. Samples for cryo sectioning were sectioned and mounted as depicted.

The labeled samples were covered with a thin layer of tissue-tek^™^ (Sakura, Copenhagen, Denmark) and immersed for 60 sec. into 2-methylbutane cooled by liquid nitrogen. The samples were immediately placed on dry ice followed by storage at -80°C until use. The samples were cut perpendicular to the surface into 30 μm slices using a cryotome (Cryotome FSE, Thermo Scientic, Demark) and transferred to coverslips (#1.5), pretreated with poly-L-Lysine (Sigma-Aldrich, Copenhagen, Denmark), as illustrated in Fig 1. They were mounted using pro-long^®^ diamond (Life technology, Naerum, Denmark) which was allowed to harden overnight before imaging.

### Labeling of skin slices with free dye solution

Untreated, unlabeled skin was cryoembedded and sectioned as described above. The sections were exposed to a 4 μM solution of Atto-488-DPPE in PBS buffer for 3 hours followed by a thorough rinsing for at least 3 x 15 min in PBS buffer.

### R**ICS microscopy setup**

RICS measurements were completed using a custom-built multiphoton excitation microscope as described in [24, 28]. In short, a 60x NA 1.29 water immersion objective was used. The laser was a Ti:Sa laser (HPeMaiTai DeepSee, Spectra Physics, Mountain View, CA), and the excitation wavelength used was 838 nm. The fluorescence signals were collected using bandpass filters ET Bandpass 572/35nm and BrightLine HC 676/29 nm (AHF Analysentechnik AG, Tübingen, Germany). The light was divided between the two detectors by a 620-DCXXR beam splitter (AHF Analysentechnik AG). The detectors were Hamamatsu H7422P-40 PMTs (Ballerup, Denmark).

### RICS and CC-RICS experiments

The RICS measurements were calibrated using 47nm green fluorescent beads Fluoro Max^™^ (Thermo Scientific, Copenhagen, Denmark). Typical measurements consist of about 50–100 fluorescence intensity images. The scan speeds and image sizes were optimized for the different diffusion coefficients in the samples. The data were analyzed using the Globals software package (Laboratory for Fluorescence Dynamics, University of California, Irvine, USA). The immobile features in the images were subtracted as described in [29]. Control measurements were carried out on unlabeled skin samples to ensure that the signal detected came from the added fluorophores and not from autofluorescence of the skin sample. The LUVs experiments have been carried out using skin from 3 different donors with >40 independent measurements. FLUVs experiments were carried out using skin from 3 different donors with >60 independent measurements.

### STED

The Atto-488-DPPE labeled samples were imaged using a Leica TSC SP8 STED setup (Mannheim, Germany). The excitation was done at 488 nm using a white light laser, and the depletion laser was a 592 nm CW. The emission was recorded at 500–580 nm using the gated hybrid detector (0.3 ns). All STED images were deconvoluted using Huygens^™^ software (Hilversum, Netherland) to further increase the resolution. STED imaging was carried out using skin from 2 different donors. The images shown are representative of >10 measurements per sample.

## Results

### Super resolution imaging of human skin

**Fig 2:**
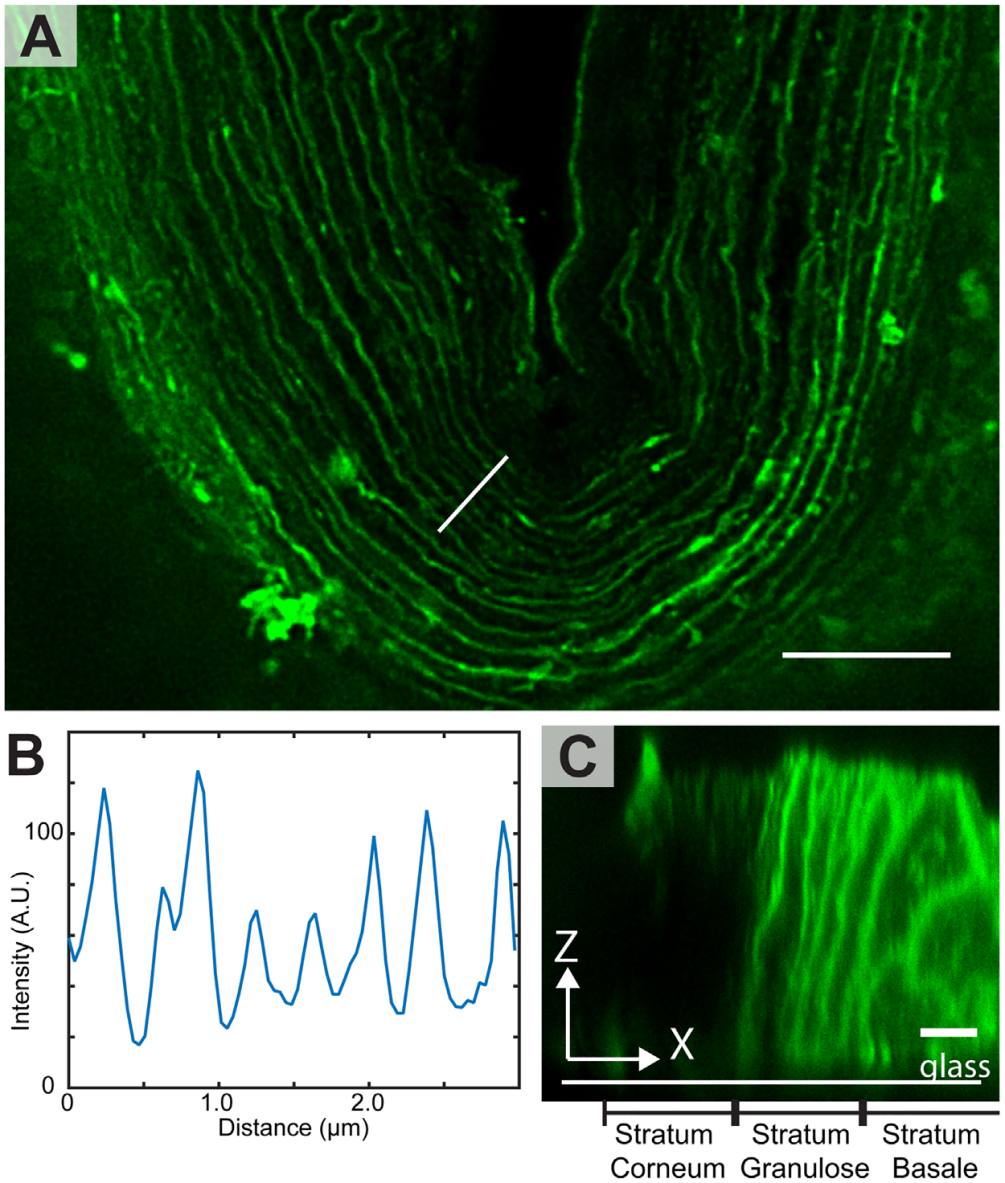
Cryo frozen and sectioned skin labelled with the free dye, Atto-488-DPPE, in PBS buffer. A) A STED image of the sample in which the SC is clearly visible, as the bright u-shaped layer in the center of the image; the width of the lipid layers in the SC are measured to approximately 100–120 nm. A line scan across the marked line is shown in B, and the individual lipid layers are recognizable. C) A confocal cross section (XZ plane) through a skin section. The slice is orientated with the SC to the left. Only the upper surface of the SC, away from the cover glass, is labeled. This is where the SC was directly exposed to the label. In contrast, the SC facing the glass (lower part in the z direction) is unlabeled. This is likely due to barrier properties of the SC towards the dye. In contrast, the SG and SS/SB are labeled all the way through in the z direction. Scale bars are 5 μm.

Human skin sections were investigated using cryo-sectioned skin, labeled with Atto-488-DPPE (without liposomes) in a PBS buffer, Fig 2. Fig 2A shows a STED image of the SC of the skin near a furrow. The different cell layers are distinguishable, and the brick and mortar structure with interchanging bright lipid layers and dark corneocytes is resolved. The width of the lipid layers are about 100–120 nm demonstrating the resolution obtainable in STED microscopy. The spacing of the lipid layers in the bottom of the furrow is about 150–200 nm and therefore could not have been resolved using traditional confocal microscopy. It is interesting to note that the SC is not labeled all the way through the section, probably due to the barrier properties of the SC toward the penetration of the dye into the tissue slice. This is evident in Fig 2C where a confocal cross section through the skin slice (XZ-plane) is shown. It can be seen that the SC (left side of Fig 2C) is only labeled on the side facing up towards the buffer with the dye, i.e. top part of Fig 2C, whereas the lower layers of the epidermis (stratum granulose (SG) and stratum basale (SB)) are labeled all the way through the section. This shows that the SC is an effective barrier to the dye even after sectioning. Intact skin samples labelled from the SC side with free dye before sectioning were also investigated using STED. Images and descriptions are presented in S2 Fig.

**Fig 3:**
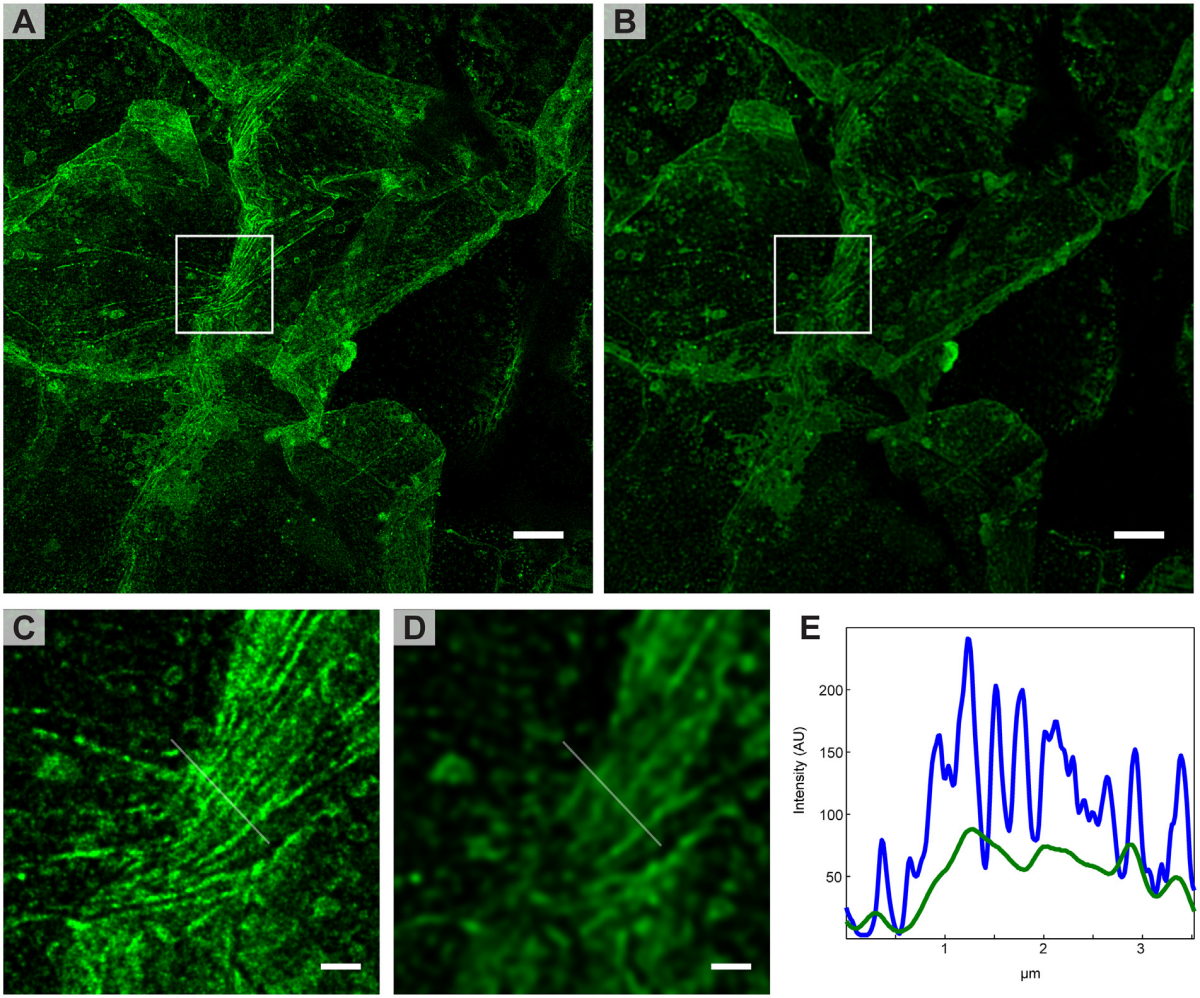
Image of the surface of intact skin. The skin samples were labeled using LUVs with Atto-488-DPPE. After labeling for 6–8 hours the samples were rinsed and patted dry, removing most of the LUVs from the surface. An intense labeling of the lipids around the corneocytes is observed. A) and B) shows a STED and confocal image, respectively, of a large area where several corneocytes can be distinguished. C) and D) are enlargements of the mark regions in A and B. The resolution difference between confocal and STED can clearly be seen. E) shows an intensity line profile across the line marked in C (blue) and D (green), again it is evident that STED reveals details not resolved in the confocal image. Both the STED and confocal image have been deconvolved. Scale bars are 5 μm for A and B, and 1 μm for C and D.

A STED and confocal image of the skin surface can be seen in Fig 3A and 3B respectively, C and D shows zooms of the marked regions. The large corneocytes are recognizable as dark regions with a bright lipid layer circumference. The marked region shows several closely packed lipid layers that are clearly resolved with STED microscopy (C) but the structures are lost in the confocal image (D). This is further shown in the intensity line profile in Fig 3E of the region marked in C and D. The FWHM of the lipid layers in the STED image are about 100 nm, demonstrating the advantage of STED microscopy over confocal microscopy.

### STED microscopy on LUVS and FLUVS in cryosectioned human skin

The FLUVs and LUVs were characterized with FCS as described in [30]. Typical vesical sizes were determined to have a diameter of 96±10nm (Data not shown). This was confirmed by STED images of the vesicles. See S1 Fig.

Fresh human skin obtained from reconstructive surgeries was exposed to LUVs and FLUVs in a non-occlusive fashion ex vivo. The STED imaging shown are from 2 different donors and are representative of >10 measurements per sample. Some samples were cryo-embedded and sectioned after exposure, while others were investigated immediately after treatment, and imaged from the skin surface of the intact, labeled skin.

**Fig 4:**
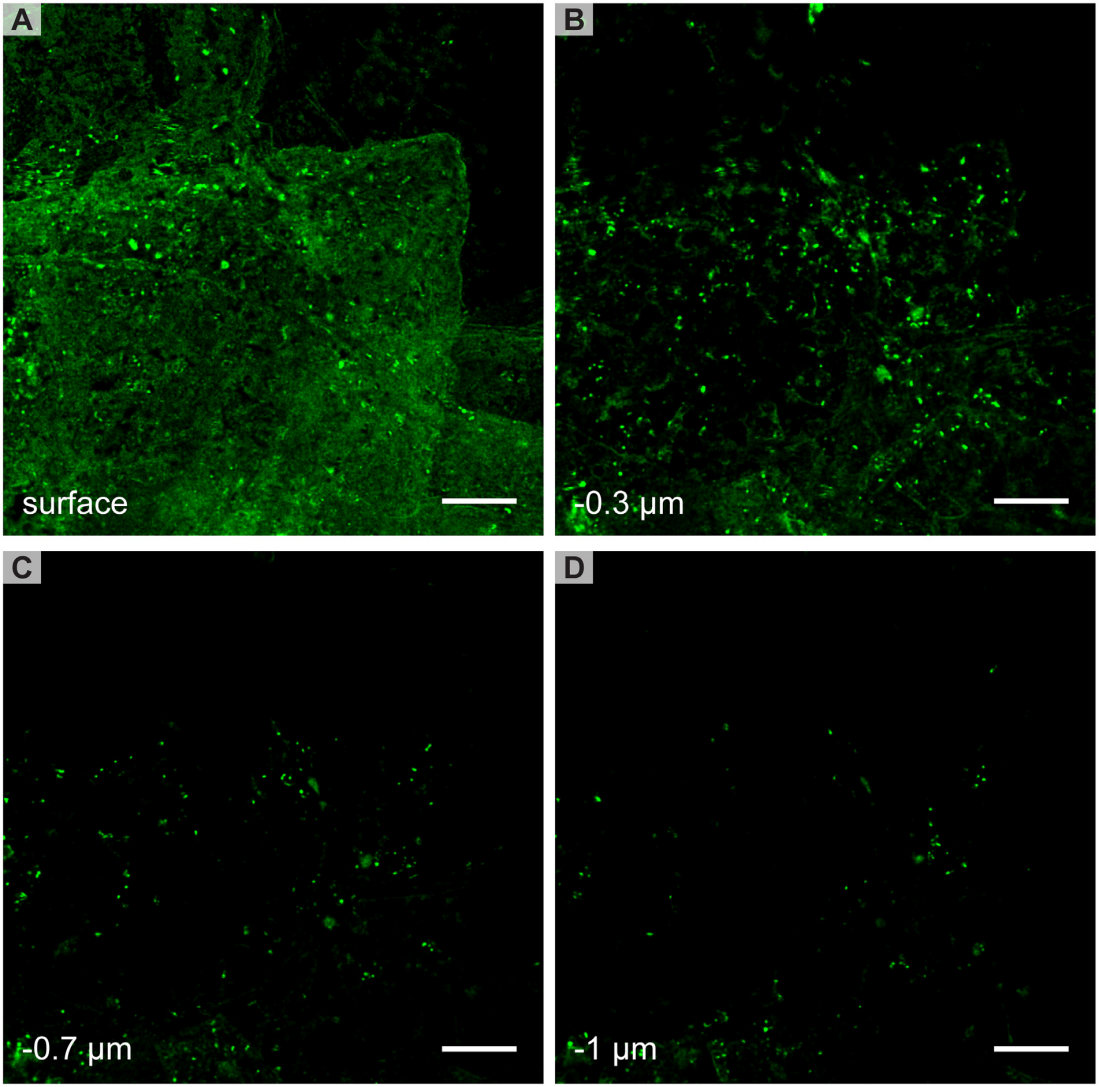
STED images of FLUVs on intact skin. Several vesicles are visible on the surface (A) and at different depths of the skin (B-D). FLUVs are not observed deeper in the skin. In the lower layers, C and D, the FLUVs are generally located at the edge of the corneocytes. Scale bars are 5 μm.

Fig 4 shows STED images of the skin at different depths after 6 hours of labeling with FLUVs. FLUVs were mainly confined to the surface of the skin. In rare cases vesicle-like structures could be seen just underneath the SC surface (>1μm), however in most cases the areas were seen to be connected to the surface due to the loose structure of the outer most SC layers. Similar results were obtained for LUVs.

**Fig 5:**
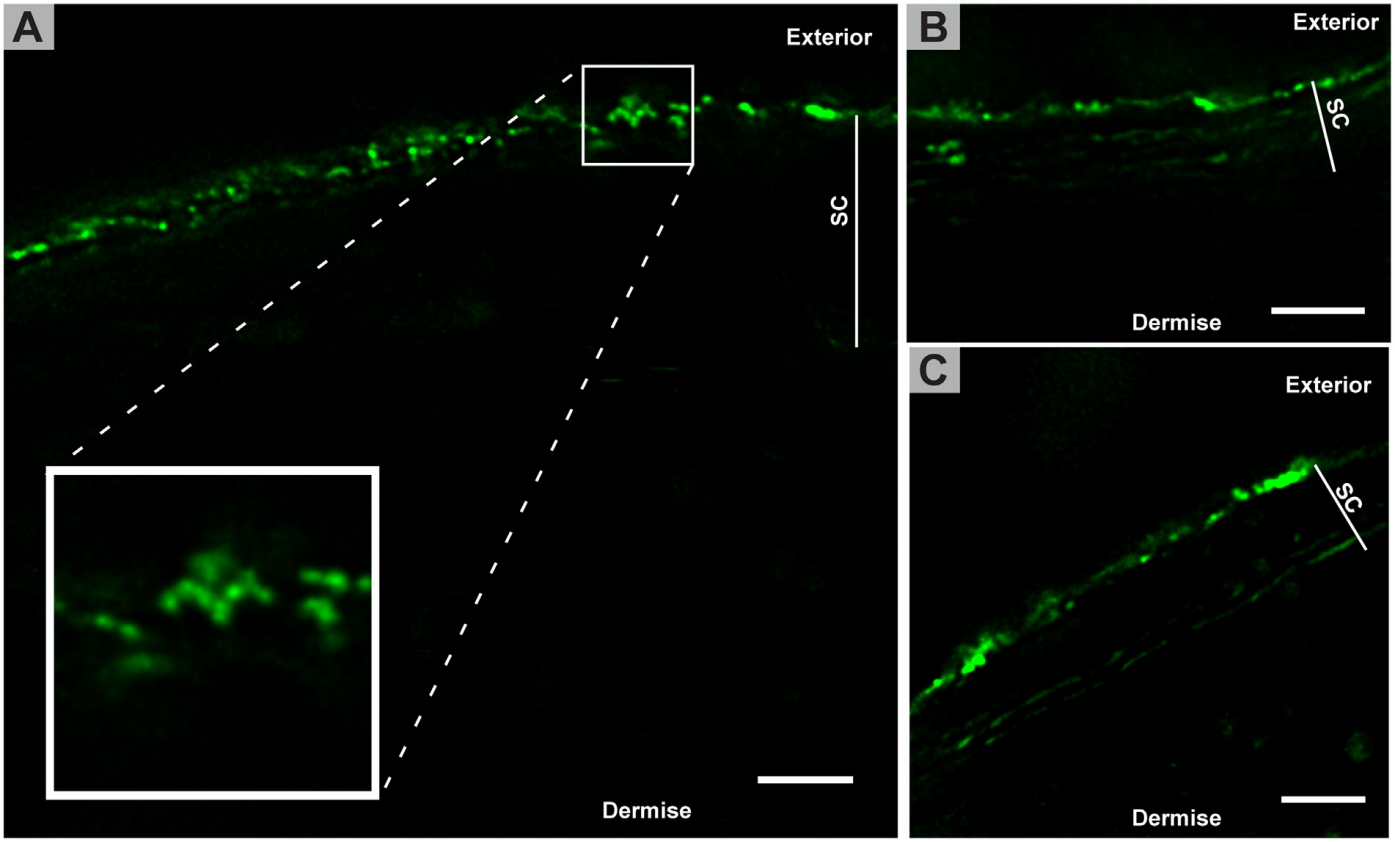
Cryoembedded and -sectioned skin exposed to POPC LUVs on the skin surface prior to processing for microscopy. Several LUVs can be seen on the surface of the SC, e.g. magnified region of interest in A. Some penetration of the dye into the superficial SC layers can be seen in B and C, where the underlying layers of the SC have become labeled by the dye included in the LUVs. LUVs are rarely found inside the SC. Scale bars are 2 μm.

**Fig 6:**
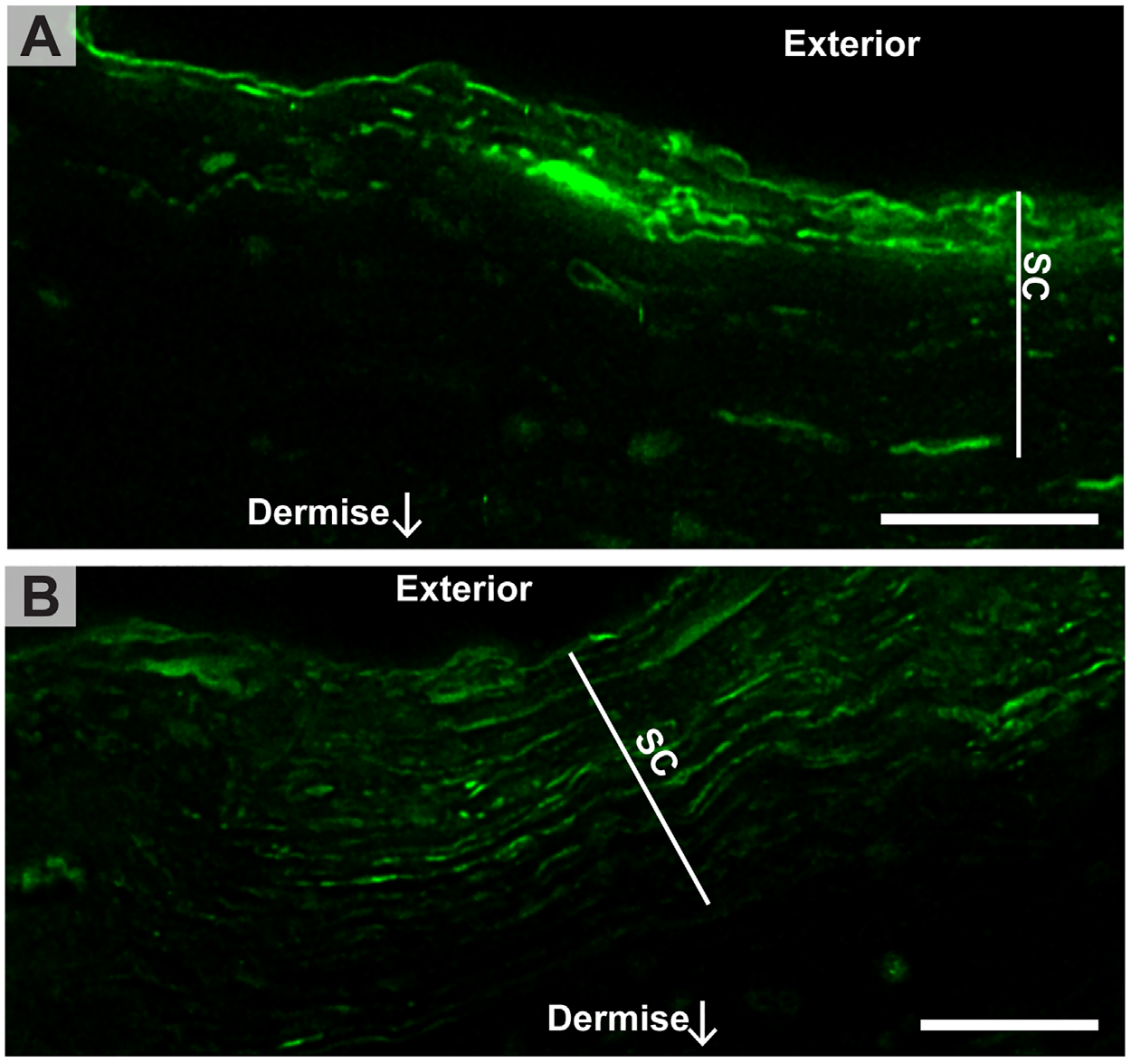
Cryoembedded and -sectioned skin exposed to FLUVs on the skin surface prior to processing for imaging. Intact FLUVs were rarely found on the surface, and the SC was generally more intensely labeled by the dye from the FLUVs than from the LUVs, indicating a better penetration of the dye from the FLUVs. Sometimes the dye was observed deeply into the SC panel B) whereas in other cases the staining was more confined to the upper most layers of SC (panel A). Scale bars are 5 μm.

Fig 5 shows images from cryosectioned samples that were exposed to LUVs prior to the cryosectioning. It is immediately clear that the LUVs have not penetrated into the skin to any great extent, and the LUVs are primarily confined to the surface. Some fluorescence is observed inside the SC (Fig 5A–5C), however, this is not confined to individual LUVs. Instead, the fluorescence is spread out over a larger area of the lipid layers in SC, indicating that the LUVs have broken or ruptured upon contact with the surface of the skin. Intact LUVs are only observed on the outermost layer of the SC. The box in Fig 5A shows a region of interest of a large cluster of several LUVs on the SC surface. Using conventional confocal microscopy, this could easily have been mistaken for a single larger unidentifiable object, but with STED microscopy, the individual LUVs can be resolved, and we provide imaging evidence for the presence of LUVs only at the SC surface. The results for the similar experiment using FLUVs can be seen in Fig 6. In general, very few intact FLUVs are observed, these would appear as bright well defined spots. Instead a more thorough labeling of the SC is seen, compared to the results with LUVs. As seen in Fig 6B, most of the SC is labeled in the samples using FLUVs, and many of the lipid layers throughout SC are visualized, but only very few vesicle-like structures (ie. having the expected size and intensity) are observed within the SC or the lower skin layers. In fact the labeling is similar to the labeling from the free dye seen in Fig 2. On the surface of the SC no intact FLUVs are seen, indicating that most of the FLUVs have ruptured on contact with the skin.

### Diffusion of FLUVs in excised human skin SC as evaluated by RICS

The CC-RICS experiments can be used to detect if two different fluorescent species of molecules move together [23, 24]. The vesicles are labeled with two different fluorophores. In intact vesicles the fluorophores are seen to move together giving a large CC-RICS signal. If the vesicles burst the diffusion of the different fluorophores will not be correlated and will resemble the diffusion of the free dyes.

**Fig 7:**
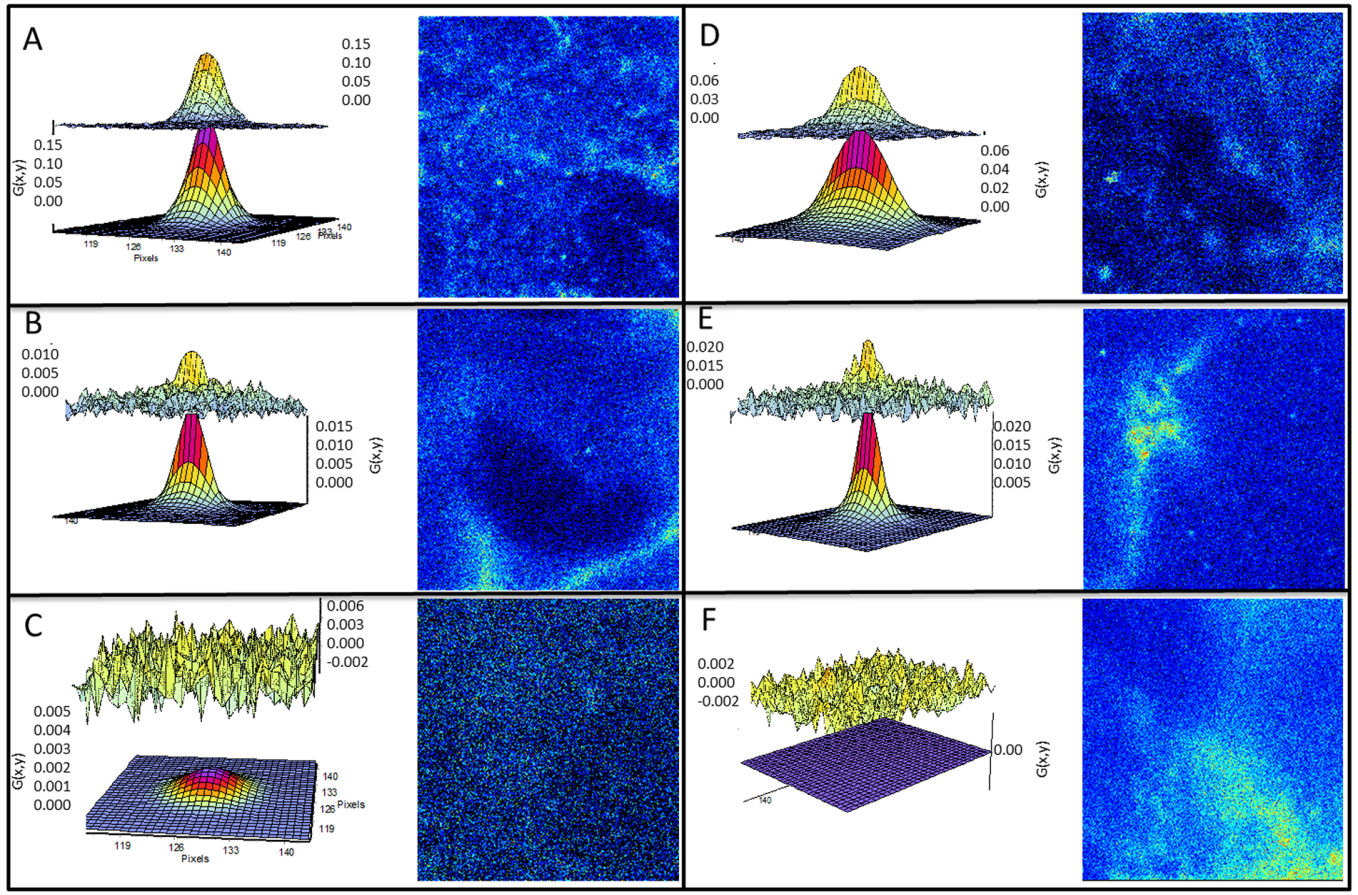
RICS data. The panels show the CC-RICS data (upper graph) and the fit (lower graph), for RhB-PE and ATTO-647N-PE labeled LUVs (panels A-C) and FLUVs (panels D-F) together with an intensity image of the ATTO-647N-PE channel. A) CC-RICS and fit for LUVs at the SC surface. A clear cross correlation is seen. B and C are CC-RICS and fit for LUVs at 4μm below the SC surface. In 15% of the measurements a cross correlation was found (panel B) while most of the measurements showed no cross correlation (panel C). D) CC-RICS and fit for FLUVs at the SC surface. A clear cross correlation is seen. E and F are CC-RICS and fit for FLUVs at 4μm below the SC surface. In 9% of the measurements a cross correlation was found (panel E) while most of the measurements showed no cross correlation (panel F). The image sizes are 22 x 22μm^2^.

The diffusion of RhB-PE and ATTO-647N-PE labeled LUVs was measured by RICS and CC-RICS in the SC of excised human skin after 4–8 hours of labeling. On the surface of the skin, the diffusion was found to be between 0.40 to 3.8 μm^2^/s for RhB-PE, ATTO-647N-PE and the cross correlation (see Fig 7A and 7D). The wide range of the diffusion values indicate the existence of a mixture of vesicles on the surface and freely diffusing vesicles on the skin surface. In nearly all of the measurements it was possible to observe a cross correlation between the fluorescence signals of the two probes. The amplitude of the correlations (G0) (which is inversely proportional to the number of fluorescent particles in the observation volume) were found to be of similar magnitude for the RhB-PE, ATTO-647N-PE and the CC-RICS.

The measurements were repeated at a depth of 3–5μm from the surface of the skin (see Fig 7B and 7C). In general the G0 value of the RhB-PE and ATTO-647N-PE was found to be quite small (under 0.01). This is due to the relatively high concentration of fluorophores in the LUVs, which made accurate measurement of the absolute values of the diffusion coefficients difficult. The diffusion coefficients were measured to be 0.5 ± 0.2 μm^2^/s for ATTO-647N-PE and 0.7± 0.3 μm^2^/s for RhB-PE. These results coincide with previously published diffusion coefficients for the free dyes in human SC; RhB-PE, 0.83±0.15μm^2^/s and ATTO-647N-PE, 0.34±0.2 μm^2^/s [24]. In about 15% of the measurements a CC was found but the distribution of the diffusion coefficient were too broad to give a quantitative measure. This indicates the coordinated diffusion of the two different dyes, which in turn indicates the existence of integral lipid bodies containing both dyes moving in the SC. Measurements under 5μm showed no CC. RICS measurements made 24 hours after labeling showed no CC.

Similar results were found for ATTO-647N-PE and RhB-PE labeled FLUVs (see Fig 7D–7F). However a cross correlation 3–5 μm under the skin was found in fewer than 9% of the measurements.

## Discussion

### STED microscopy in human SC

Techniques such as TEM [12, 13, 31] and different types of optical microscopy including laser scanning microscopy (LSM) [9, 16, 18, 19] and MPEM [30, 32, 33] have been used to image skin. While the TEM images deliver the highest resolution, they have the drawback of a rather invasive sample preparation, low specificity, and difficulty in interpreting the images [34]. On the other hand LSM and MPEM can be used on intact skin tissue and can have high specificity due to labeling with specific fluorophores. However, they suffer from low resolution (200–300 nm) due to the diffraction limit. The STED microscopy images of the SC (see Fig 2) show that the enhanced resolution of STED can resolve details like the lipid layers in extracellular space of the SC, which would not be visible in normal LSM or MPEM. This demonstrates that STED is a valuable new tool for examining the skin structure and for bridging the gap between TEM and LSM or MPEM images. However, STED does have some drawbacks that are closely related to those of LSM and MPEM, namely that the resolution becomes worse with increasing depth into the sample and the necessity of using a fluorescent label.

### STED and RICS on LUVs and FLUVs in human SC

The RICS and CC-RICS data obtained in this study reveals several interesting features. For the measurements on or near the surface of the skin the LUVs and FLUVs behave similarly, and a range of diffusion constants are measured. The higher values correspond well with free diffusion of a particle in water of about 100 nm, where Stoke-Einstein’s equation for diffusion gives a diffusion constant of about 4 μm^2^/s. The slower diffusion constant is either caused by vesicles diffusing along the surface of the skin or absorbing or dispersing onto the skin surface, which is a process that will affect the RICS data [24]. The fact that the two types of vesicles behave similarly on the surface is reasonable considering that they have the same size. It is worth noting that after the non-occlusive incubation a large number of the vesicles of either type are still found, typically in furrows on the surface.

The RICS measurements of the LUVs and FLUVs below 5μm inside the skin gave no cross correlation indicating that no intact vesicles penetrate below this depth. The fact that a reservoir of vesicles is still available on the surface and that vesicles are not seen to penetrate suggests that the penetration is not a fast process followed by bursting as we do not see any fast moving vesicles in the SC.

The RICS measurements performed at depths between 3–5μm inside the skin give diffusion coefficients for the two different fluorophores which are very close to the measurements we have presented previously for free dyes diffusing inside the skin [24]. However, for both mixtures of liposomes, doubly labeled with dyes, a cross correlation was found in 15% of LUVs and 9% of FLUVs measurements, which indicates the presence of lipid bodies (intact or fragmented) containing both dyes. This result coincides with multiple studies using TEM on multiple vesicle types [12, 13]. The CC-RICS data do not take into account the number of burst vesicles also present on the surface, and in principle only a few intact vesicles among many burst vesicles would still give a positive cross correlation.

It is important to note that the values of 15% and 9% does not mean that 15% of the LUVs and 9% of the FLUVs penetrate the SC. Considering that the concentration of labeled liposomes added to the sample was in the order of 1 nM (measured by RICS) we calculated that more than10^10^ liposomes per cm^2^ of the skin were delivered during the incubations. Therefore, the vesicles observed inside the SC (down to 4 μm) are only a small fraction of those applied. Below 5 μm, no cross correlation was found. Therefore we consider that the cross correlations most likely arise from small patches of membrane or vesicles co-diffusing, in the upper areas of the SC with some direct contact to the skin surface.

The CC-RICS results match well with the STED images where most of the fluorescence was found on the outermost layer of the SC. On the surface, the STED images showed that LUVs frequently could be found as vesicles, whereas the FLUVs were rarely found intact on the surface. This was not due to the cryoembedding and sectioning as the vesicles were not destroyed by this processing (see S1 Fig).

In general, very few intact LUVs or FLUVs could be identified in the SC. However, there are some striking differences between the two types of vesicles. The LUVs seem to be more stable, and were more likely to maintain a vesicle-like shape on the skin surface as seen in Fig 3, however only a small amount of the fluorescence probe was found inside the skin. In contrast to this, intact FLUVs were rarely seen on surface of the skin, but there was a significantly increased amount of fluorescence labeling in the deeper layers of the SC of the samples treated with FLUVs. The STED images showed that the fluorescent signal was not found in any vesicle-like shapes, indicating that most of the vesicles had burst or otherwise lost their integrity. This result provides evidence that the FLUVs act as permeability enhancer that promote the uptake of the fluorophore into the skin.

The proposed transdermal penetration of FLUVs has previously been described to occur by two different mechanisms: The first being that the FLUVs being ultra-flexible can squeeze their way through the SC and act as a drug carrier system, the second mechanism being that the FLUVs function as chemical penetration enhancers. Cevc et al reported intact drug loaded FLUVs in the blood stream of mice after treatment with fluorescently labeled vesicles [17], and others have found penetration of loaded vesicles through frozen abdominal porcine skin from intrapartum stillborn animals using Franz diffusion cells[35]. On the other hand, others, using human skin, have suggested that the penetration of intact liposomes into the human epidermis is highly unlikely due to low amounts of vesicle material found in the deeper layers of the SC [36]. It is important to remember that there is a significant difference between skin from animal models and humans. For example mouse SC is much thinner than human SC and therefore the barrier properties are not readily comparable [37, 38]. Our results strongly indicate that it is highly unlikely for FLUVs or LUVs to penetrate the human SC as intact liposomes, as we saw no cross correlation and no vesicles below 5μm in the SC. We have previously shown that the diffusion of dual-color fluorescently labeled liposomes—containing an amphiphilic fluorophore in the lipid bilayer and a hydrophilic fluorophore encapsulated in the liposome lumen vesicles—showed no cross-correlation between the fluorophores below the skin surface, indicating that the penetration of intact liposomes is highly compromised by the skin barrier [24]. Together with the work presented in this paper, the combined results suggest that the main mechanism of action for LUVs or FLUVs is not that they penetrate intact through the skin and deliver their cargo as proposed by Cevc et al. and Honneywell-Nguyen et al. [14, 39]. Rather, our results suggest that most of the FLUVs burst on the surface of the skin, and that they enhance the delivery through the SC by another mechanism. The POPC LUVs show a lower delivery through the SC compared with FLUVs. This can be ascribed to the stability/rigidity of the LUVs which could indicate that they do not interact as strongly with the SC as the flexible FLUVs. It is also possible that the difference in the components in the FLUVs, specifically the detergent sodium cholate, could mediate an increased fusion or partitioning of the FLUV with the SC lipid membranes. Therefore we conclude that the mechanism of action for the FLUVs is more likely to be related to chemical disruption of the lipid layers in the SC, as has previously been reported for several detergents and liposomes [15, 28, 40, 41].

## Supporting Information

S1 Fig Characterization of LUV and FLUV. (DOCX)

S2 Fig Investigation of labeled skin slices. (DOCX)
