# Supplementary material for: Superresolution and Fluorescence Dynamics Evidence Reveal That Intact Liposomes Do Not Cross the Human Skin Barrier
Source: PLoS One. 2016 Jan 11;11(1):e0146514. doi: 10.1371/journal.pone.0146514 (PMC4709185; doi:10.1371/journal.pone.0146514)
Supplement: S1 Fig — (DOCX) [file pone.0146514.s001.docx]

## Characterization of LUV and FLUV


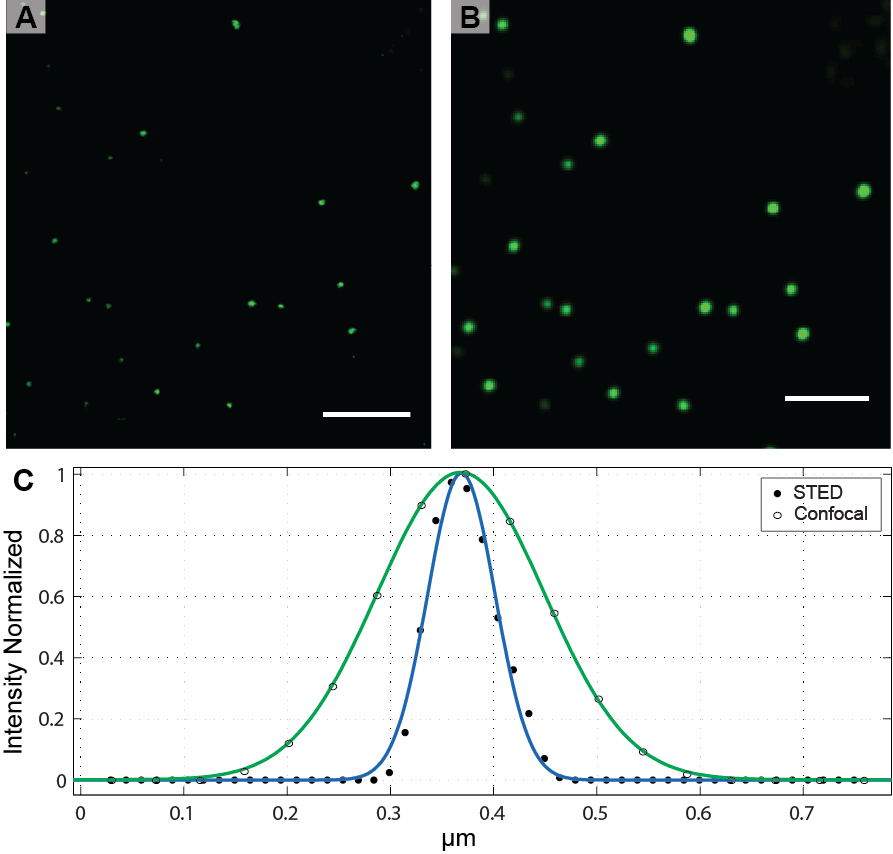


**S1** **Fig.** FLUV imaged after the cryo frezzing process and cutting. The FLUV were trapped in the cryo media outside the skin. The FLUV were imaged with both STED (A) and confocal (B) microscopy. A line scan across a typical FLUV is shown in C for both STED (filled) and confocal (hollow) and a Gaussion fit, blue and green respectively, is also shown. The FWHM for each fit is 109 nm for the STED and 270 nm for confocal. The scale bars are 2 µm

Both the FLUV and LUV were characterized thoroughly in terms of determine their size and monodispersity using FCS and RICS. The fabrication procedure is a thoroughly tested procedure in our lab, and previous characterization was confirmed. For a thorough description we refer to our previously publication with the FLUV and LUV [1]. I short the LUV were measured with a diffusion coefficient of $3.20\pm0.2 \mu m^{2}/s$ corresponding to a diameter of 134±8, using Stokes-Einstein equation, likewise the FLUV were measured to 120±20 nm in diameter, matching well with reports from other labs[2-4]. The LUV and FLUV were also investigated after the cryo-freezing as is shown in Fig. S1. Here FLUV were investigated post-freezing and slicing using both conventional confocal and STED microscopy. In the confocal image, Fig. S1B, the FLUV looks very similar and have a size of about 270 nm, as shown in Fig. S1C, however the STED image, Fig. S1A, is able to fully resolve the FLUV and shows the dispersion in their sizes, a line scan of one of the FLUV shows a diameter of 109 nm, corresponding well with the FCS and RICS measurements done pre-freezing and -slicing.

[1] J. Brewer, M. Bloksgaard, J. Kubiak, J.A. Sorensen, L.A. Bagatolli, Spatially Resolved Two-Color Diffusion Measurements in Human Skin Applied to Transdermal Liposome Penetration, J Invest Dermatol, 133 (2013) 1260-1268.

[2] M.E.M.J. van Kuijk-Meuwissen, H.E. Junginger, J.A. Bouwstra, Interactions between liposomes and human skin in vitro, a confocal laser scanning microscopy study, Biochimica et Biophysica Acta (BBA) - Biomembranes, 1371 (1998) 31-39.

[3] G. Cevc, Drug delivery across the skin, Expert opinion on investigational drugs, 6 (1997) 1887-1937.

[4] G. Cevc, U. Vierl, Nanotechnology and the transdermal route A state of the art review and critical appraisal, Journal of Controlled Release, 141 (2010) 277-299.
