# Supplementary material for: Superresolution and Fluorescence Dynamics Evidence Reveal That Intact Liposomes Do Not Cross the Human Skin Barrier
Source: PLoS One. 2016 Jan 11;11(1):e0146514. doi: 10.1371/journal.pone.0146514 (PMC4709185; doi:10.1371/journal.pone.0146514)
Supplement: S2 Fig — (DOCX) [file pone.0146514.s002.docx]

## Investigation of labeled skin slices


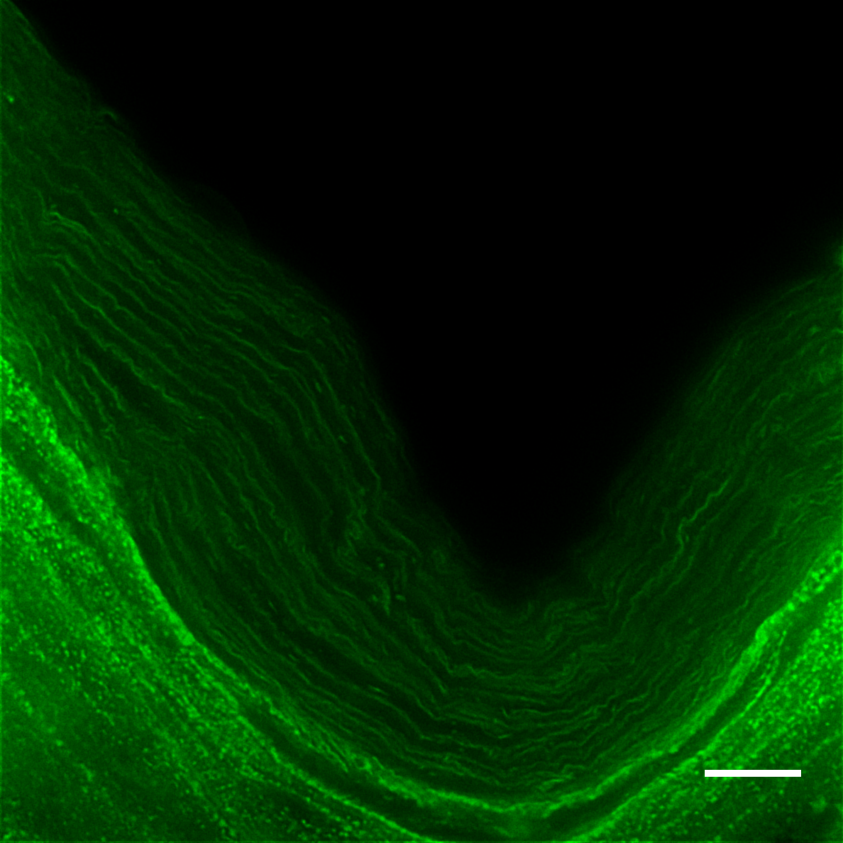


**S2 Fig.** Skin slices labeled with Atto-488-DPPE dissolved in DMSO. Several of the lipid layers in the SC can be measured to be about 100 nm showing the resolution obtainable in STED microscopy. Scale bar is 5 µm.

To get a better labeling of the SC a skin slice was also labeled with Atto-488-DPPE dissolved in DMSO, which dramatically enhances the penetration of the dye in the SC. However as is also evident in the image, the SB are not labeled well using DMSO. Several of lipid layers in the SC can be measured to be about 100 nm showing the resolution obtainable in these skin tissue samples.
